# Supplementary material for: Plasma ACE2 predicts outcome of COVID-19 in hospitalized patients
Source: PLoS One. 2021 Jun 4;16(6):e0252799. doi: 10.1371/journal.pone.0252799 (PMC8177449; doi:10.1371/journal.pone.0252799)
Supplement: S1 Fig — A. Day 0 plasma ACE2 in hospitalized COVID-19 positive patients divided in outcome groups 2 (intubated) or 3–4 (not intubated) for day 0 study window (enrollment plus 24 hours). B. Day 3 plasma abundance of ACE2 in hospitalized COVID-19 positive patients divided in outcome groups 2 (intubated) or 3–4 (not intubated) for day 3 study window. C. Day 7 plasma ACE2 in hospitalized COVID-19 positive patients divided in outcome groups 2 (intubated) or 3–4 (not intubated) for day 7 study window. The acuity groups: 2 = Intubated, ventilated. 3 = Hospitalized, supplementary O2 required. 4 = Hospitalized, no supplementary O2 required. Bars indicate median and interquartile range. Data were analyzed using the Mann Whitney test. ** P< 0.01. **** P < 0.0001. (DOCX) [file pone.0252799.s001.docx]

**S1 Fig.**

**S1 Fig. Plasma ACE2 in hospitalized COVID-19 positive patients with samples analyzed at all time points divided in clinical outcome groups 2 (intubated) or 3-4 (not intubated). A.** Day 0 plasma ACE2 in hospitalized COVID-19 positive patients divided in outcome groups 2 (intubated) or 3-4 (not intubated) for day 0 study window (enrollment plus 24 hours). **B.** Day 3 plasma abundance of ACE2 in hospitalized COVID-19 positive patients divided in outcome groups 2 (intubated) or 3-4 (not intubated) for day 3 study window. **C.** Day 7 plasma ACE2 in hospitalized COVID-19 positive patients divided in outcome groups 2 (intubated) or 3-4 (not intubated) for day 7 study window. The acuity groups: 2 = Intubated, ventilated. 3 = Hospitalized, supplementary O2 required. 4 = Hospitalized, no supplementary O2 required. Bars indicate median and interquartile range. Data were analyzed using the Mann Whitney test. ** P< 0.01. **** P < 0.0001.
